# Virtual Reality Enhanced Exercise Training in Upper Limb Function of Patients With Stroke: Meta-Analytic Study

**DOI:** 10.2196/66802

**Published:** 2025-02-19

**Authors:** Shiqi Xu, Yanwen Xu, Ruyi Wen, Jun Wang, Yuyu Qiu, Chetwyn CH Chan

**Affiliations:** 1 Department of Rehabilitation Medicine Wuxi Ninth People’s Hospital Affiliated to Soochow University Wuxi, Jiangsu China; 2 Wuxi School of Medicine Jiangnan University Wuxi, Jiangsu China; 3 School of Rehabilitation Medicine Nanjing Medical University Nanjing China; 4 Department of Psychology The Education University of Hong Kong Hong Kong SAR China

**Keywords:** virtual reality, stroke, upper limb function, exercise training, meta-analysis

## Abstract

**Background:**

Recovery of upper limb function after stroke secondary to ischemia or hemorrhage is crucial for patients’ independence in daily living and quality of life. Virtual reality (VR) is a promising computer-based technology designed to enhance the effects of rehabilitation; however, the results of VR-based interventions remain equivocal.

**Objective:**

This study aims to review the plausible factors that may have influenced VR’s therapeutic effects on improving upper limb function in patients with stroke, with the goal of synthesizing an optimal VR intervention protocol.

**Methods:**

The databases PubMed, EMBASE, Web of Science, and Cochrane Library were queried for English-language papers published from May 2022 onward. Two reviewers independently extracted data from the included papers, and discrepancies in their findings were resolved through consensus during joint meetings. The risk of bias was assessed using the Physiotherapy Evidence Database Scale and the Methodological Index for Non-Randomized Studies. Outcome variables included the Action Research Arm Test, Box-Block Test, Functional Independence Measure, Upper Extremity Fugl-Meyer Assessment, and Wolf Motor Function Test. The plausible factors examined were age, total dosage (hours), trial length (weeks), session duration (hours/session), frequency (sessions/week), and VR content design. The Bonferroni adjustment was applied to *P* values to prevent data from being incorrectly deemed statistically significant.

**Results:**

The final sample included 15 articles with a total of 1243 participants (age range 48.6-75.59 years). Participants in the VR therapy (VRT) group (n=455) demonstrated significantly greater improvements in upper limb function and independence in activities of daily living compared with those in the conventional therapy group (n=301). Significant factors contributing to improved outcomes in upper limb function were younger age (mean difference [MD] 5.34, 95% CI 2.18-8.5, *P*<.001; I2=0%), interventions lasting more than 15 hours (MD 9.67, 95% CI 4.19-15.15, *P*<.001; I2=0%), trial lengths exceeding 4 weeks (MD 4.02, 95% CI 1.39-6.65, *P*=.003; I2=15%), and more than 4 sessions per week (MD 3.48, 95% CI 0.87-6.09, *P*=.009; I2=0%). However, the design of the VR content, including factors such as the number of features (eg, offering exercise and functional tasks; individualized goals; activity quantification; consideration of comorbidities and baseline activity level; addressing patient needs; aligning with patient background such as education level; patient-directed goals and interests; goal setting; progressive difficulty levels; and promoting self-efficacy), did not demonstrate significant effects (MD 3.89, 95% CI –6.40 to 1.09; effect Z=1.36, *P*=.16).

**Conclusions:**

Greater VR effects on improving upper limb function in patients with stroke were associated with higher training doses (exceeding 15 hours) delivered over 4-6 weeks, with shorter sessions (approximately 1 hour) scheduled 4 or more times per week. Additionally, younger patients appeared to benefit more from the VR protocol compared with older patients.

## Introduction

Recovery of upper limb function after stroke is crucial for patients’ independent daily living and quality of life [1-3]. Previous studies have demonstrated the positive effects of incorporating neurodevelopmental, proprioceptive neuromuscular, and motor relearning theories in designing interventions to improve patients’ upper limb function [4-7]. Advances in technology have enabled virtual reality (VR) to serve as a common medium for delivering interventions aimed at enhancing upper limb function [8]. VR is a promising computer technology that allows users to interact with a simulated multisensory environment [9]. It is designed to enhance the effectiveness of rehabilitation and provide feedback on performance. However, a review of clinical studies examining the use of VR as an intervention for patients with stroke produced inconsistent findings. Compared with usual practice, VR interventions were found to be more effective in improving upper-extremity and hand functions in patients with stroke [10-12]. By contrast, 1 study reported that VR interventions were effective in improving cognitive functions but not motor functions in patients with traumatic brain injury [13], while another study found no significant differences between VR and conventional therapy for patients with stroke [14].

A review of recent studies suggested that the inconsistent results described above may stem from variations in the design, content, duration, and intensity of VR interventions [14], as well as patient-specific characteristics such as independence level and emotional state [12]. For example, VR exercise training was found to be more effective than usual practice for improving upper limb function in patients with stroke only when they participated in a high-dose program (eg, 15 hours or more) [15], whereas this effect was not observed with a low-dose program (eg, 12 hours) [16]. In the high-dose study, the VR exercise training included a variety of limb movements with varying levels of difficulty. Conversely, the low-dose study used a VR protocol consisting of 3 modes of hand and arm movements performed at different speeds. Inconsistent findings are also evident in studies evaluating the effects of VR in patients with Parkinson’s disease. For example, a VR game using a 1-group pre- and posttest design demonstrated improvements in hand grip strength, dexterity, and speed among patients with stroke [17]. The VR game comprised 4 activities delivered in sequence: the reach game, the sequence game, the grab game, and the flip game. The intervention involved 30-minute individual sessions conducted 3 times per week over 6 weeks (18 sessions, totaling 9 hours). These results contrast with those of a randomized controlled trial (RCT), which found no significant effects [18]. The RCT protocol involved tracking and touching moving objects displayed on a screen while maintaining a standing balance. The schedule consisted of 40-minute individual sessions, 3 sessions per week over 6 weeks (18 sessions, totaling 12 hours). Many national clinical guidelines for stroke emphasize the importance of applying recovery principles. Beyond dosage (ie, duration per session, frequency, and overall intervention period), the design of VR training content appears to influence treatment outcomes. For instance, the 2023 edition of the National Clinical Guideline for Stroke for the United Kingdom and Ireland [19] recommends therapy for motor recovery and function that incorporates individualized content, a variety of practice modes, intensity, and the practice of functional skills. Additionally, studies have highlighted the critical role of repetitions in reshaping neuronal structures and enhancing motor system function following brain injury [20-22]. Research on nonprimates has shown that performing repeated reaching tasks can induce synaptogenesis and changes in cortical representations [23,24]. In human studies, evidence suggests that functional recovery of the upper limbs, driven by the brain’s plasticity, requires at least 300 repetitions per day [25] or 100 active movements or more per treatment session [26].

The inconsistent findings regarding the effects of VR-based interventions motivated us to undertake this meta-analytic study. We aimed to examine 6 plausible factors that might influence the therapeutic effects of VR on improving upper limb function in patients with stroke. These factors included patients’ age, dosage (hours), delivery schedule (session duration, frequency, and trial length), total dosage (hours), and content design. We hypothesized that all these factors would significantly impact intervention outcomes. Identifying the factors contributing to variability in intervention outcomes would enhance the content design of VR interventions and provide guidance on setting appropriate dosages and schedules to achieve better treatment results for patients.

## Methods

### Design

This study followed the scope and methods outlined in the PRISMA (Preferred Reporting Items for Systematic Reviews and Meta-Analyses) statement and its accompanying checklist [27].

### Search Strategy

The relevant materials for review and analysis were extracted on May 7, 2022, from PubMed (1966 to present), EMBASE via Ovid (1974 to present), Web of Science (1956 to present), and the Cochrane Library databases (no date restriction). The medical terms and free-text search terms were related to VR, upper limb, and rehabilitation. Detailed search strategies for each database are provided in Multimedia Appendix 1.

### Inclusion and Exclusion Criteria

The inclusion and exclusion criteria set for the studies are presented in Textboxes 1 and 2.

**Inclusion criteria.**

- Randomized controlled trial and group comparison studies.
- Results published in English in peer-reviewed journals.
- The treatment group used virtual reality–enhanced exercise alone or combined with conventional therapy, while the control group used conventional therapy alone or without treatment.
- Patients showed upper limb functional disabilities with outcome variables including the Action Research Arm Test, Box-Block Test, Functional Independence Measure, Upper Extremity Fugl-Meyer Assessment, and Wolf Motor Function Test.
- Means, SD, and effect sizes were presented; or means, SEs, *t* values, or *P* values were presented; or range or 95% CIs were presented.

**Exclusion criteria.**

- Abstracts, case reports, or review studies.
- Non-English or nonbilingual journal articles.
- Research design without group comparison.
- Patients without stroke or measuring lower limb functions of patients with stroke.
- Missing data or additional information (means, SD, and effect sizes) cannot be obtained from the corresponding author within 1 month after initial contact.

### Study Selection

All studies were imported into the EndNote referencing software (Thomson Research Soft, version X9), and duplicates were identified and removed. Two reviewers, SX and JW, co-authors of this paper, independently conducted the relevance screening. The screening process began with an evaluation of the title and abstract content of each publication based on the inclusion and exclusion criteria outlined above. Reviewers were instructed to read the full text if the abstract contained ambiguous content. Discrepancies between the 2 reviewers were resolved through consensus during joint meetings.

### Data Extraction

Two research team members (SX and YX) independently extracted data from the included papers. For each paper, the researchers identified the trials and specific content, recording them in preformatted tables. The recorded information included trial design and setup, sample sizes of all groups, intervention program details, overall characteristics, and clinical outcome results. Discrepancies between the 2 reviewers were resolved through consensus during joint meetings.

### Assessment of Risk of Bias

Two research team members (YX and YQ) independently assessed the risk of bias in the included trials using the Physiotherapy Evidence Database (PEDro) Scale [28] and the Methodological Index for Non-Randomized Studies (MINORS) [29]. The 11-item PEDro Scale evaluates the quality of the trials, with a score of “1” indicating the criterion is met and “0” indicating it is not. PEDro quality grades are categorized as high (6-8: good; 9-10: excellent), fair (4-5: acceptable), and poor (≤4). For nonrandomized trials, the first 8 items of the MINORS were used to assess methodological quality (risk of bias). Each MINORS item is scored as follows: “0” for not reported, “1” for reported but insufficient, and “2” for reported and sufficient. For a single-group trial, the maximum score is 16, while for a 2-group trial, it is 24. A score of 0-9 for a single-group trial indicates poor quality and 10-16 indicates high quality. In this study, the cut-off for inclusion was set at high quality or above: a score of 6 or higher on the PEDro Scale or 10 or higher on the MINORS [30]. Discrepancies in the scores assigned by the 2 reviewers were resolved through consensus during joint meetings.

The principles of therapy content (motor recovery and function) outlined in the 2023 edition of the National Clinical Guideline for Stroke for the United Kingdom and Ireland [19] were adopted as feature criteria to guide the analysis of VR content. Ten content features were identified: offering exercise and functional tasks; setting individualized goals; quantifying activity; considering comorbidities and baseline activity levels; incorporating patient needs; matching the patient’s background, such as education level; aligning with the patient’s goals and interests; involving goal setting; including progressive difficulty levels; and promoting self-efficacy. The content features of each study were collated and classified into 3 categories: “+” for few features (2-3), “++” for medium features (4-5), and “+++” for many features (≥6).

### Data Synthesis and Analysis

We conducted analyses using Review Manager (RevMan) version 5.3 software. As some studies [14,31-34] reported results in terms of the median, minimum, maximum, or IQR, we converted these to means and SDs as proposed in the study by Wan et al [35]. For studies reporting only means, SEs, *t* values, or *P* values, we computed the SDs [31,32,35]. For studies reporting only ranges or 95% CIs, we calculated the means [31,32,35]. Statistical heterogeneity for each meta-analysis was assessed using the Cochran *Q* test and the *I*^2^ statistic. Fixed-effect models were applied when heterogeneity among trials was nonsignificant (*I*^2^<50%), while random-effect models were used for significant heterogeneity (*I*^2^>50%). The outcome variables included the Action Research Arm Test (ARAT), Box and Block Test (BBT), Functional Independence Measure (FIM), Upper Extremity Fugl-Meyer Assessment (FMA-UE), and Wolf Motor Function Test (WMFT). All results were reported as mean differences (MDs) with 95% CIs. The plausible factors considered were age (with the World Health Organization defining older age as 60 years and above), total dosage (hours), trial length (weeks), duration of a session (hours/session), frequency (sessions/week), and VR content design. The cut-off value was set based on the findings of previous literature [36-39] and the principle that at least two articles must be included per subgroup. As only 15 studies were available for analysis, multiple comparisons were conducted separately for each of the 6 factors to meet this criterion. Statistical significance for the meta-analysis (whole group) was set at *P*<.05. For the subgroup analyses, the potential proliferation of type I and type II errors across the 6 comparisons was controlled by applying the Bonferroni adjustment, setting statistical significance at *P*<.008 (0.05 divided by 6).

## Results

### Study Selection

**Figure 1:**
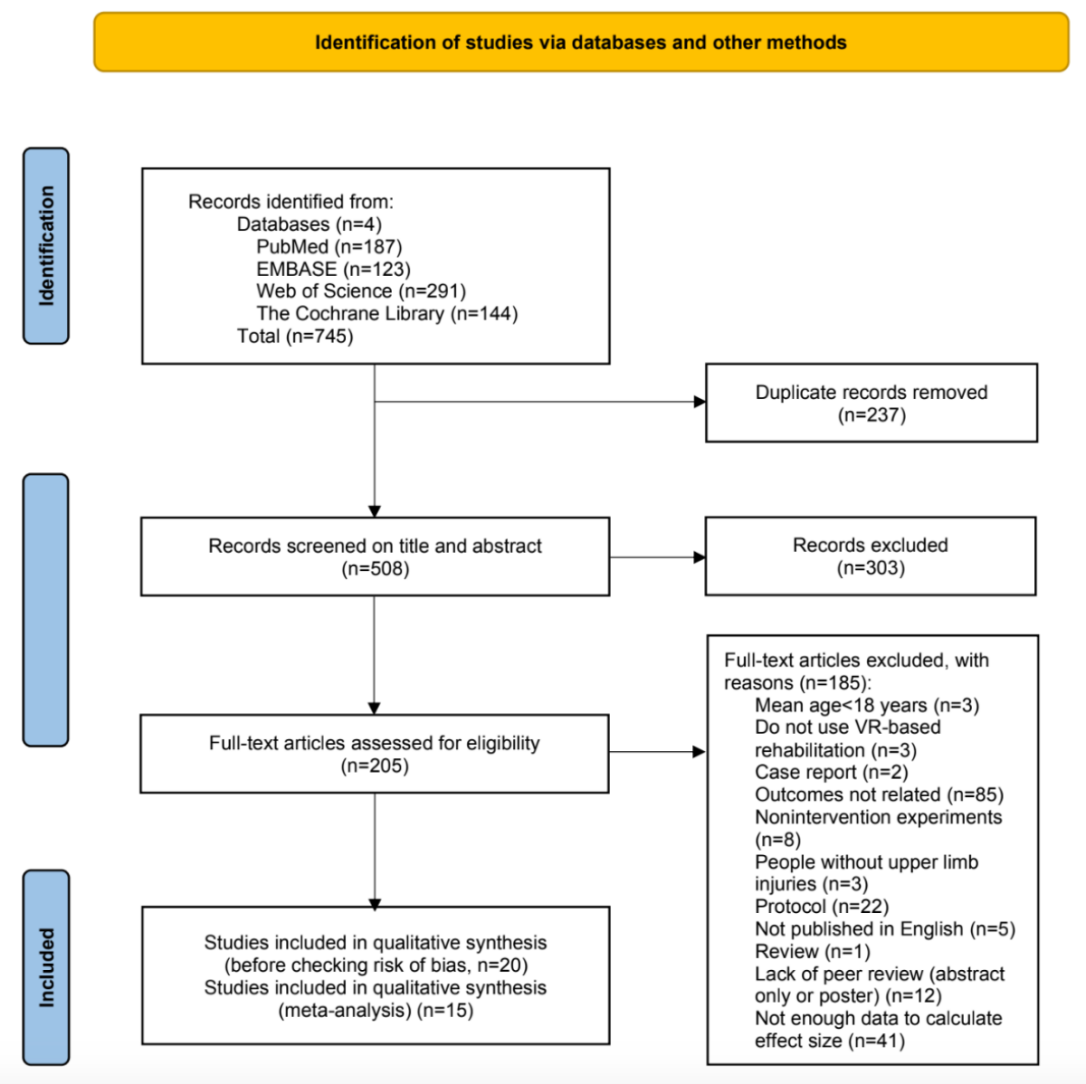
PRISMA (Preferred Reporting Items for Systematic Reviews and Meta-Analyses) flowchart showing 15 records entering the analyses. VR: virtual reality.

Details of the steps taken to select the studies are presented in the PRISMA flowchart (Figure 1; also see Multimedia Appendix 2). A total of 745 studies were retrieved from 4 databases through an electronic search. Of these, 237 studies were excluded due to duplication. An additional 303 studies were excluded after reviewing their titles and abstract contents. Full-text screening of the remaining 205 studies resulted in the exclusion of 185 studies for not meeting the inclusion criteria. Among the remaining 20 studies, 5 were further excluded for being rated below the “good” cut-offs on the PEDro and MINORS scales. The final number of studies included in the analyses was 15 [11,16,33,34,40-50], consisting of 12 RCTs and 3 non-RCTs.

### Characteristics of the Included Trials

The 15 studies included a total of 28 groups, comprising 15 VR therapy (VRT) groups and 13 conventional therapy (CON) groups, with 1203 participants in total (Table 1). All studies were published between 2013 and 2022. At the total group level (14 studies), the VRT and CON groups included 411/278 and 294/177 males/females, respectively. One study did not report gender as a grouping variable and was therefore excluded from the gender analysis at the group level. The average ages of participants in the VRT groups ranged from 49.0 to 73.0 years, while those in the CON groups ranged from 53.4 to 75.6 years. The intervention durations for the VRT groups varied from 2 weeks to 3 months. Fourteen studies [11,16,33,34,40-47,49,50] involved patients with stroke secondary to ischemia or hemorrhage, while 1 study [48] included only patients with stroke secondary to ischemia.

**Table 1:** Characteristics of the trials contained in 15 studies satisfying the inclusion criteria.

Study	Study design	Participants	Age (years), mean	Intervention	Control	Dosage	Total dosage (hours)	Outcomes included in this review
		Intervention/control (no/no)	Intervention/control (yes/yes)					
Brunner et al [40]	RCT^a^	62/58	62/62	YouGrabber system	Conventional training	60 minutes/day, 4 days/week, 4 weeks	16	ARAT^b^, BBT^c^, and FIM^d^
Abd El-Kafy et al [41]	RCT	20/20	54.13/53.42	Conventional physiotherapy and training with robot-mediated VR^e^ gaming	Conventional physiotherapy	2 hours/day, 3 days/week, 12 weeks	72	ARAT and WMFT^f^
Gueye et al [42]	RCT	25/25	66.56/68.12	Armeo Spring	Conventional physiotherapy	45 minutes/day, 4 days/week, 3 weeks	9	FMA-UE^g^ and FIM
Hsu et al [11]	RCT	18/17	52.9/56.9	VR-based mirror therapy and usual care	Conventional occupational therapy and usual care	50 minutes/day, 2 days/week, 9 weeks	15	FMA-UE and BBT
Kiper et al [43]	RCT	23/21	64.3/64.3	VR rehabilitation system and traditional rehabilitation	Traditional rehabilitation	2 hours/day, 5 days/week, 4 weeks	40	FMA-UE and FIM
Kiper et al [44]	RCT	68/68	62.5/66	VR rehabilitation system and traditional rehabilitation	Traditional rehabilitation	2 hours/day, 5 days/week, 4 weeks	40	FMA-UE and FIM
Rong et al [45]	RCT	20/20	56.25/62.3	Camera-based mirror visual feedback and robot-assisted training	Sham-mirror visual feedback and robot-assisted training	1.5 hours/day, 5 days/week, 4 weeks	30	FMA-UE and FIM
Schuster-Amft et al [16]	RCT	22/32	61.3/61.2	VR-based training system	Conventional therapy	45 minutes/day, 4 days/week, 4 weeks	12	BBT
Sin and Lee [46]	RCT	18/17	71.78/75.59	Xbox Kinect and conventional occupational therapy	Conventional occupational therapy	1 hour/day, 3 days/week, 6 weeks	18	FMA-UE and BBT
Taveggia et al [47]	RCT	27/27	73/68	Armeo spring and conventional treatment	Conventional treatment	1 hour/day, 5 days/week, 6 weeks	30	FIM
Yao et al [48]	RCT	20/20	63/66.2	C-tDCS^h^ and VR therapy and conventional occupational and physical therapies	Sham tDCS and VR and conventional occupational and physical therapies	20 minutes/day, 5 days/week, 2 weeks	3.33	ARAT and FMA-UE
Zheng et al [49]	RCT	58/54	65.4/66.2	Low-frequency repetitive transcranial magnetic stimulation and VR and standard rehabilitation therapy for stroke	Sham repetitive transcranial magnetic stimulation and VR training and standard rehabilitation therapy for stroke	2 hours/day, 6 days/week, 4 weeks	48	WMFT
Turolla et al [50]	Non-RCT	263/113	60.2/65.4	Upper limb conventional and reinforced feedback in the virtual environment therapies	Upper limb conventional therapy	2 hours/day, 5 days/week, 4 weeks	40	FMA-UE and FIM
Borstad et al [33]	Non-RCT	16/N/A^i^	49/N/A	Constraint-induced movement therapy (recovery rapids and highly trained practitioners)	N/A	3 hours/day, 5 days/week, 2 weeks	30	ARAT and WMFT
Sebastián-Romagosa et al [34]	Non-RCT	51/N/A	60.52/N/A	Brain-computer interface systems	N/A	1 hour/day, 2 days/week, 3 months	25	FMA-UE

^a^RCT: randomized controlled trial.

^b^ARAT: Action Research Arm Test.

^c^BBT: Box-Block Test.

^d^FIM: Functional Independence Measure.

^e^VR: virtual reality.

^f^WMFT: Wolf Motor Function Test.

^g^FMA-UE: Upper Extremity Fugl-Meyer Assessment.

^h^C-tDCS: cathodal transcranial direct current stimulation.

^i^N/A: not applicable.

### Risk of Bias

The mean PEDro Scale score for the 12 studies with a randomized clinical trial design was 7.2 (Table 2). All these studies reported the method of randomization, baseline comparability, blinding of outcome assessors, participant dropout rates of less than 15%, between-group outcome analysis, and point estimates with variability. Most of these studies also reported adequate assignment concealment (n=7, 58%) and intention-to-treat analyses (n=4, 33%). Among the studies with a nonrandomized clinical trial design (n=3), it is noteworthy that they demonstrated clear research objectives and prospective data collection but lacked a follow-up period and did not perform sample size calculations (Table 3).

**Table 2:** Physiotherapy Evidence Database Scale score for randomized clinical trials included in the review.

Studies	Total score	Methodological quality	Number of items of the Physiotherapy Evidence Database Scale										
			1^a^	2^b^	3^c^	4^d^	5^e^	6^f^	7^g^	8^h^	9^i^	10^j^	11^k^
Ikbali Afsar et al [14]	5	Fair	N/A^l^	✓^m^	N/A	✓	N/A	N/A	✓	N/A	N/A	✓	✓
Alves et al [10]	5	Fair	N/A	✓	N/A	✓	N/A	N/A	✓	N/A	N/A	✓	✓
Brunner et al [40]	7	Good	N/A	✓	✓	✓	✓	N/A	N/A	✓	N/A	✓	✓
Abd El-Kafy et al [41]	6	Good	N/A	✓	✓	✓	N/A	N/A	✓	✓	N/A	✓	✓
Ersoy and Iyigun [51]	4	Poor	N/A	✓	✓	✓	N/A	N/A	N/A	N/A	N/A	✓	✓
Gueye et al [42]	6	Good	N/A	✓	✓	✓	N/A	N/A	N/A	✓	✓	✓	✓
Hsu et al [11]	7	Good	N/A	✓	✓	✓	N/A	N/A	✓	✓	N/A	✓	✓
Junior et al [52]	5	Fair	N/A	✓	N/A	✓	N/A	N/A	✓	N/A	N/A	X	X
Kiper et al [43]	6	Good	N/A	✓	N/A	✓	N/A	N/A	✓	✓	N/A	✓	✓
Kiper et al [44]	8	Good	N/A	✓	✓	✓	N/A	N/A	✓	✓	✓	✓	✓
Rong et al [45]	8	Good	N/A	✓	✓	✓	N/A	N/A	✓	✓	✓	✓	✓
Schuster-Amft et al [16]	8	Good	N/A	✓	✓	✓	N/A	✓	✓	✓	N/A	✓	✓
Sin and Lee [46]	7	Good	N/A	✓	N/A	✓	N/A	✓	✓	✓	N/A	✓	✓
Taveggia et al [47]	7	Good	N/A	✓	✓	✓	✓	N/A	N/A	✓	N/A	✓	✓
Yao et al [48]	8	Good	N/A	✓	✓	✓	N/A	✓	✓	✓	N/A	✓	✓
Zheng et al [49]	8	Good	N/A	✓	N/A	✓	N/A	✓	✓	✓	✓	✓	✓

^a^Item 1: Specified eligibility criteria.

^b^Item 2: Random allocation.

^c^Item 3: Concealed allocation.

^d^Item 4:Baseline comparability.

^e^Item 5: Participants were blinded.

^f^Item 6: Therapists were blinded.

^g^Item 7: Assessors were blinded.

^h^Item 8: Adequate follow-up.

^i^Item 9: Intention-to-treat analysis.

^j^Item 10: Between-group comparisons.

^k^Item 11: Point estimates and variability.

^l^N/A: not applicable.

^m^The ‘✓’ symbol indicates that the item where it is found has been punctuated.

**Table 3:** Methodological Index for Non-Randomized Studies Scale score for nonrandomized clinical trials included in the review.

Studies	Total score	Methodological quality	Number of items of the Methodological Index for Non-Randomized Studies Scale^a^											
			1^b^	2^c^	3^d^	4^e^	5^f^	6^g^	7^h^	8^i^	9^j^	10^k^	11^l^	12^m^
Turolla et al [50]	15/24	Good	2	2	2	2	2	0	0	0	2	2	0	1
Borstad et al [33]	10/16	Good	2	2	2	2	1	1	0	0	N/A	N/A	N/A	N/A
Kizony et al [53]	7/16	Poor	2	1	2	2	0	0	0	0	N/A	N/A	N/A	N/A
Sebastián-Romagosa et al [34]	10/16	Good	2	2	2	2	1	1	0	0	N/A	N/A	N/A	N/A

^a^The items are scored 0 (not reported), 1 (reported but inadequate), or 2 (reported and adequate). The global ideal score is 16 for noncomparative studies and 24 for comparative studies.

^b^Item 1: A clearly stated aim.

^c^Item 2: Inclusion of consecutive patients.

^d^Item 3: Prospective collection of data.

^e^Item 4: Endpoints appropriate to the aim of the study.

^f^Item 5: Unbiased assessment of the study endpoint.

^g^Item 6: Follow-up period appropriate to the aim of the study.

^h^Item 7: Loss to follow-up less than 5%.

^i^Item 8: Prospective calculation of the study size.

^j^Item 9: An adequate control group.

^k^Item 10: Contemporary groups.

^l^Item 11: Baseline equivalence of groups.

^m^Item 12: Adequate statistical analyses.

### Meta-Analysis

#### Whole-Group: Upper Limb Function (FMA-UE)

**Figure 2:**
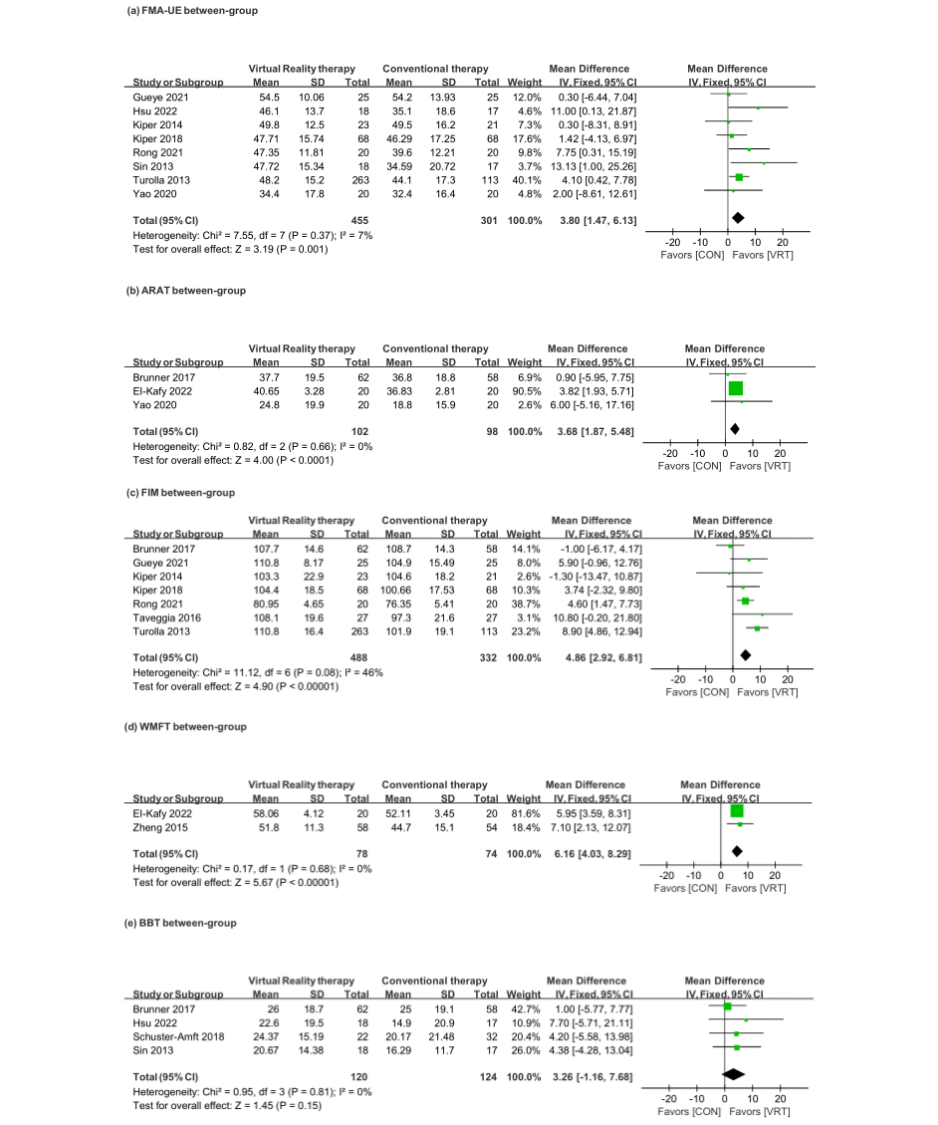
Forest plot of comparisons of between-group outcomes on upper limb function. CON: conventional therapy; VRT: virtual reality therapy.

**Figure 3:**
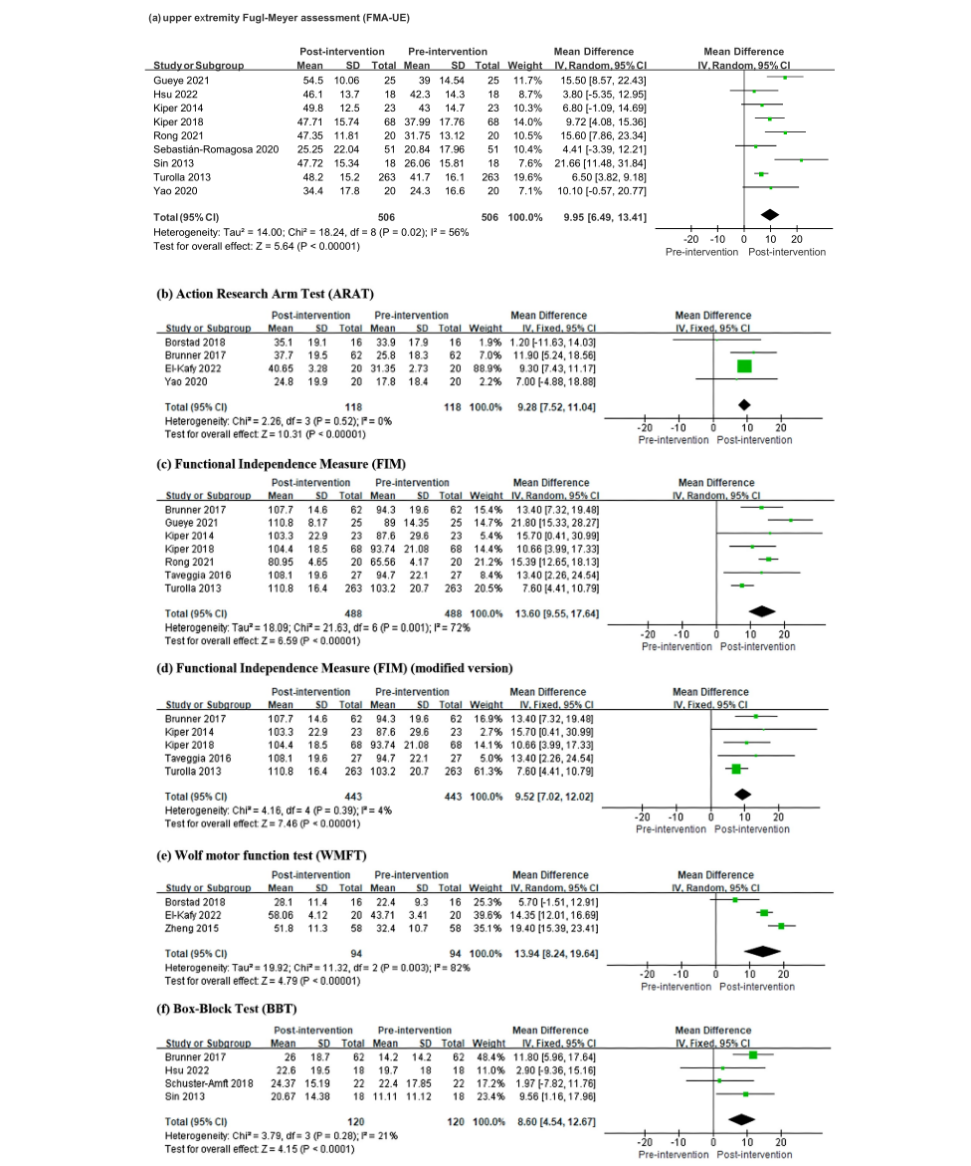
Forest plot of comparisons of within-group outcomes on upper limb function.

Participants in the VRT group (n=455) demonstrated significantly greater improvement than those in the CON group (n=301) in FMA-UE scores (MD 3.80, 95% CI 1.47-6.13, *P*=.001; *I*^2^=7%; Figure 2; see also [11,16,40-50]). Within the VRT group, participants showed significant increases in FMA-UE scores following the intervention (MD 9.95, 95% CI 6.49-13.41, *P*<.001; *I*^2^=56%; Figure 3; see also [11,16,33,34,40-50]).

#### Whole Group: Upper Limb Activity Limitation (ARAT, FIM, WMFT, and BBT)

Participants in the VRT group showed significantly greater improvements than those in the CON group in upper limb function measured by the ARAT (MD 3.68, 95% CI 1.87-5.48, *P*<.001; *I*^2^=0%; n [VRT]=102, n [CON]=98; Figure 2), independence in activities of daily living measured by the FIM (MD 4.86, 95% CI 2.92-6.81, *P*<.001; *I*^2^=46%; n [VRT]=488, n [CON]=332; Figure 2), and upper limb functional capacity measured by the WMFT (MD 6.16, 95% CI 4.03-8.29, *P*<.001; *I*^2^=0%; n [VRT]=78, n [CON]=74; Figure 2). However, there were no significant differences between the groups in upper limb finger dexterity measured by the BBT (MD 3.26, 95% CI –1.16 to 7.68, *P*=.15; *I*^2^=0%; n [VRT]=120, n [CON]=124; Figure 2).

Among the VRT groups, participants demonstrated significant pre- and postintervention improvements in scores on the ARAT (MD 9.28, 95% CI 7.52-11.04, *P*<.001; *I*^2^=0%), FIM (MD 13.60, 95% CI 9.55-17.64, *P*<.001; *I*^2^=72%; or MD 9.52, 95% CI 7.02-12.02, *P*<.001; *I*^2^=4% after the removal of outliers [21,25]), WMFT (MD 13.94, 95% CI 8.24-19.64, *P*<.001; *I*^2^=82%), and BBT (MD 8.60, 95% CI 4.54-12.67, *P*<.001; *I*^2^=21%; Figure 3).

### Subgroup Analyses

#### Age Influencing the VR Effect

Compared with those in the CON group, younger patients (n=301, mean age 56.45 years) in the VRT group demonstrated significantly greater improvements in upper limb function measured by the FMA-UE (MD 5.34, 95% CI 2.18-8.5, *P*<.001; Table 4). No statistically significant heterogeneity was observed among older patients (SMD 1.96, 95% CI −1.50 to 5.41, *P*=.27; Table 4). Similarly, younger patients showed greater improvements in independence in activities of daily living measured by the FIM (MD 6.55, 95% CI 2.36-10.75, *P*=.002), while no statistically significant heterogeneity was observed among older patients (MD 2.68, 95% CI −0.47 to 5.83, *P*=.10; Table 5).

**Table 4:** Subgroup analyses on age, total hours, trial length, and duration based on FMA-UEa measures.

Outcomes, moderating factors, and between-group/within-group comparison						Number of trials analyzed and number of participants involved			Mean difference (95% CI)			*P* value			*I*^2^ (%)		
**FMA-UE**																	
	**Age (years)**																
		**Younger (≤60.52 years)**															
			Between-group	3; N (VRT^b^)=301, N (CON^c^)=150			5.34 (2.18 to 8.50)			.0009			0				
			Within-group	4; N=352			6.96 (4.64 to 9.29)			<.001			48				
		**Older (** **>** **60.52 years)**															
			Between-group	5; N (VRT)=154, N (CON)=151			1.96 (–1.50 to 5.41)			.27			0				
			Within-group	5; N=154			11.93 (8.54 to 15.33)			<.001			42				
	**Total dosage (hours)**																
		**<** **15**															
			Between-group	2; N (VRT)=45, N (CON)=45			0.79 (–4.90 to 6.47)			.79			0				
			Within-group	2; N=45			13.90 (8.09 to 19.71)			<.001			0				
		≥**15 and ≤30**															
			Between-group	3; N (VRT)=56, N (CON)=54			9.67 (4.19 to 15.15)			.0005			0				
			Within-group	4; N=107			11.14 (2.99 to 19.30)			<.001			72				
		**>** **30**															
			Between-group	3; N (VRT)=354, N (CON)=202			2.95 (0.06 to 5.84)			.05			0				
			Within-group	3; N=354			7.07 (4.76 to 9.38)			<.001			0				
	**Trial length (weeks)**																
		**<** **4**															
			Between-group	2; N (VRT)=45, N (CON)=45			0.79 (–4.90 to 6.47)			.79			0				
			Within-group	2; N=45			13.90 (8.09 to 19.71)			<.001			0				
		≥**4 and ≤6**															
			Between-group	5; N (VRT)=392, N (CON)=239			4.02 (1.39 to 6.65)			.003			15				
			Within-group	5; N=392			10.88 (6.07 to 15.68)			<.001			67				
		**>** **6**															
			Between-group	1; N (VRT)=18, N (CON)=17			11.0 (0.13 to 21.87)			.05			N/A^d^				
			Within-group	2; N=69			4.15 (–1.78 to 10.09)			.17			0				
	**Duration of a session (hours)**																
		**<** **1**															
			Between-group	2; N (VRT)=45, N (CON)=45			0.79 (–4.90 to 6.47)			.79			0				
			Within-group	2; N=45			13.90 (8.09 to 19.71)			<.001			0				
		≥**1 and** **<2**															
			Between-group	3; N (VRT)=56, N (CON)=54			9.67 (4.19 to 15.15)			.0005			0				
			Within-group	4; N=107			11.14 (2.99 to 19.30)			<.001			72				
		≥**2**															
			Between-group	3; N (VRT)=354, N (CON)=202			2.95 (0.06 to 5.84)			.05			0				
			Within-group	3; N=354			7.07 (4.76 to 9.38)			<.001			0				
	**Frequency (days/week)**																
		≤**4**															
			Between-group	3; N (VRT)=61, N (CON)=59			7.01 (–1.67 to 15.68)			.11			58				
			Within-group	4; N=112			11.17 (3.14 to 19.20)			.006			73				
		**>** **4**															
			Between-group	5; N (VRT)=394, N (CON)=242			3.48 (0.87 to 6.09)			.009			0				
			Within-group	5; N=394			7.86 (5.69 to 10.03)			<.001			27				

^a^FMA-UE: Upper Extremity Fugl-Meyer Assessment.

^b^VRT: virtual reality therapy.

^c^CON: conventional therapy.

^d^N/A: not applicable.

**Table 5:** Subgroup analyses on age, total hours, trial length, and duration based on the Functional Independence Measure.

Outcomes, moderating factors, and between-group/within-group comparison					Number of trials analyzed and number of participants involved		Mean difference (95% CI)		*P* value		*I*^2^ (%)
**Functional Independence Measure**											
	**Age (years)**										
		**Younger (≤60.52)**									
			Between-group	2; N (VRT^a^)=283, N (CON^b^)=133		6.55 (2.36 to 10.75)		.002		63	
			Within-group	2; N=283		11.54 (3.91 to 19.17)		.003		92	
		**Older (** **>** **60.52)**									
			Between-group	5; N (VRT)=205, N (CON)=199		2.68 (–0.47 to 5.83)		.10		26	
			Within-group	5; N=205		15.13 (11.72 to 18.55)		<.001		36	
	**Total dosage (hours)**										
		**<30**									
			Between-group	2; N (VRT)=87, N (CON)=83		2.07 (–4.65 to 8.79)		.55		60	
			Within-group	2; N=87		17.52 (9.29 to 25.75)		<.001		71	
		≥**30 and <40**									
			Between-group	2; N (VRT)=47, N (CON)=47		5.06 (2.06 to 8.07)		.001		11	
			Within-group	2; N=47		15.28 (12.62 to 17.94)		<.001		0	
		≥**40**									
			Between-group	3; N (VRT)=354, N (CON)=202		6.70 (3.46 to 9.94)		<.001		46	
			Within-group	3; N=354		8.43 (5.6 to 11.26)		<.001		0	
	**Duration of a session (hours)**										
		**<** **1**									
			Between-group	1; N (VRT)=25, N (CON)=25		5.90 (–0.96 to 12.76)		.09		N/A^c^	
			Within-group	1; N=25		21.80 (15.33 to 28.27)		<.001		N/A	
		≥**1 and <2**									
			Between-group	3; N (VRT)=109, N (CON)=105		3.54 (–1.60 to 8.68)		.18		61	
			Within-group	3; N=109		14.98 (12.54 to 17.41)		<.001		0	
		≥**2**									
			Between-group	3; N (VRT)=354, N (CON)=202		6.70 (3.46 to 9.94)		<.001		46	
			Within-group	3; N=354		8.43 (5.60 to 11.26)		<.001		0	
	**Frequency (days/week)**										
		≤**4**									
			Between-group	2; N (VRT)=87, N (CON)=83		2.07 (–4.65 to 8.79)		.55		60	
			Within-group	2; N=87		17.52 (9.29 to 25.75)		<.001		71	
		**>4**									
			Between-group	5; N (VRT)=401, N (CON)=249		5.82 (3.62 to 8.03)		<.001		26	
			Within-group	5; N=401		11.89 (7.31 to 16.47)		<.001		71	

^a^VRT: virtual reality therapy.

^b^CON: conventional therapy.

^c^N/A: not applicable.

#### Dosage (Total Hours) Influencing the VR Effect

Comparisons were conducted between studies with total intervention delivery times of 15 hours or less and those exceeding 15 hours. Among studies with less than 15 hours of intervention, the VRT group did not show significant changes in upper limb function, as measured by the FMA-UE, compared with the CON group before and after the intervention (MD 0.79, 95% CI −4.90 to 6.47, *P*=.79; Table 4). By contrast, among studies with over 15 hours of intervention, the VRT group demonstrated significant improvements in upper limb function measured by the FMA-UE (MD 9.67, 95% CI 4.19-15.15, *P*<.001; Table 4).

#### Trial Length Influencing the VR Effect

When the trial duration was 4 weeks or longer, improvements in upper limb function measured by the FMA-UE in the VRT group were greater than those in the CON group (MD 4.02, 95% CI 1.39-6.65, *P*<.001; Table 4). For within-group comparisons, trials lasting over 4 weeks showed significant improvements in upper limb function in the VRT group measured by the ARAT (MD 9.49, 95% CI 7.69-11.29, *P*<.001; Table 6) and the BBT (MD 7.43, 95% CI 0.50-14.36, *P*=.04; Table 7). The VRT group also demonstrated improvements in upper limb function measured by the FMA-UE when the trial duration was less than 6 weeks (MD 10.88, 95% CI 6.07-15.68, *P*<.001; Table 4), but no significant improvements were observed when the trial duration was 6 weeks or longer (MD 4.15, 95% CI −1.78 to 10.09, *P*=.17; Table 4).

**Table 6:** Subgroup analyses on age, total hours, trial length, and duration based on Action Research Arm Test measures.

Outcomes and moderating factors				Between-group/within-group comparison		Number of trials analyzed and number of participants involved		Mean difference (95% CI)		*P* value		*I*^2^ (%)
**Action Research Arm Test**												
	**Age (years)**											
		Younger (≤60.52)	Within-group		2; N=36		9.13 (7.28 to 10.98)		<.001		33	
		Older (>60.52)	Within-group		2; N=82		10.73 (4.92 to 16.54)		<.001		0	
	**Total dosage (hours)**											
		<30	Within-group		2; N=82		10.73 (4.92 to 16.54)		<.001		0	
		≥30	Within-group		2; N=36		9.13 (7.28 to 10.98)		<.001		33	
	**Trial length** **(weeks)**											
		<4	Within-group		2; N=36		4.32 (–4.39 to 13.04)		.33		0	
		≥4	Within-group		2; N=82		9.49 (7.69 to 11.29)		<.001		0	
	**Duration of a session (hours)**											
		≤1	Within-group		2; N=82		10.73 (4.92 to 16.54)		<.001		0	
		>1	Within-group		2; N=36		9.13 (7.28 to 10.98)		<.001		33	
	**Frequency (days/week)**											
		≤4	Within-group		2; N=82		9.49 (7.69 to 11.29)		<.001		0	
		>4	Within-group		2; N=36		4.32 (–4.39 to 13.04)		.33		0	

**Table 7:** Subgroup analyses on age, total hours, trial length, and duration based on Box-Block Test measures.

Outcomes, moderating factors, and between-group/within-group comparison					Number of trials analyzed and number of participants involved		Mean difference (95% CI)		*P* value		*I*^2^ (%)
**Box-Block Test**											
	**Age (years)**										
		**Younger (≤60.52)**									
			Between-group	1; N (VRT^a^)=18, N (CON^b^)=17		7.70 (–5.71 to 21.11)		.26		N/A^c^	
			Within-group	1; N=18		2.90 (–9.36 to 15.16)		.64		N/A	
		**Older (** **>** **60.52)**									
			Between-group	3; N (VRT)=102, N (CON)=107		2.72 (–1.96 to 7.40)		.25		0	
			Within-group	3; N=102		9.31 (5.00 to 13.62)		<.001		30	
	**Total dosage (hours)**										
		≤**15**									
			Between-group	2; N (VRT)=40, N (CON)=49		5.42 (–2.49 to 13.32)		.18		0	
			Within-group	2; N=40		2.33 (–5.32 to 9.98)		.55		0	
		**>** **15**									
			Between-group	2; N (VRT)=80, N (CON)=75		2.28 (–3.05 to 7.62)		.4		0	
			Within-group	2; N=80		11.07 (6.27 to 15.87)		<.001		0	
	**Trial length (weeks)**										
		≤**4**									
			Between-group	2; N (VRT)=84, N (CON)=90		2.04 (–3.53 to 7.60)		.47		0	
			Within-group	2; N=84		7.70 (–1.80 to 17.20)		.11		65	
		**>** **4**									
			Between-group	2; N (VRT)=36, N (CON)=34		5.36 (–1.92 to 12.63)		.15		0	
			Within-group	2; N=36		7.43 (0.50 to 14.36)		.04		0	
	**Duration of a session (hours)**										
		**<1**									
			Between-group	2; N (VRT)=40, N (CON)=49		5.42 (–2.49 to 13.32)		.18		0	
			Within-group	2; N=40		2.33 (–5.32 to 9.98)		.55		0	
		≥**1**									
			Between-group	2; N (VRT)=80, N (CON)=75		2.28 (–3.05 to 7.62)		.4		0	
			Within-group	2; N=80		11.07 (6.27 to 15.87)		<.001		0	
	**Frequency (days/week)**										
		**<** **4**									
			Between-group	2; N (VRT)=36, N (CON)=34		5.36 (–1.92 to 12.63)		.15		0	
			Within-group	2; N=36		7.43 (0.50 to 14.36)		.04		0	
		≥**4**									
			Between-group	2; N (VRT)=84, N (CON)=90		2.04 (–3.53 to **7**.60)		.47		0	
			Within-group	2; N=84		7.70 (–1.80 to 17.20)		.11		65	

^a^VRT: virtual reality therapy.

^b^CON: conventional therapy.

^c^N/A: not applicable.

#### Duration (Hour/Session) and Frequency (Session/Week) Influencing the VR Effect

For session durations shorter than 2 hours, participants in the VRT group (mean 1.17 hours) demonstrated significantly greater improvements in upper limb function, as measured by the FMA-UE, compared with those in the CON group (mean 1.17 hours) (MD 9.67, 95% CI 4.19-15.15, *P*<.001; Table 4). For session durations equal to or longer than 2 hours (ie, ≥2 hours/session), participants in the VRT group (mean 2.0 hours) also showed significantly greater improvements in independence in activities of daily living, as measured by the FIM, compared with those in the CON group (mean 2.0 hours; MD 6.70, 95% CI 3.46-9.94, *P*<.001; Table 5).

When the frequency exceeded 4 sessions per week, improvements in upper limb function measured by the FMA-UE (MD 3.48, 95% CI 0.87-6.09, *P*<.001) and independence in activities of daily living measured by the FIM in the VRT group were greater than those in the CON group (MD 5.82, 95% CI 3.62-8.03, *P*<.001) (Tables 4 and 5). Conversely, when the frequency was 4 or fewer sessions per week, significant changes in upper limb function measured by the ARAT were observed in the VRT group (MD 9.49, 95% CI 7.69-11.29, *P*<.001), but no significant changes were found when the frequency exceeded 4 sessions per week (MD 4.32, 95% CI –4.39 to 13.04, *P*=.33; Table 6).

#### Content Design Influencing the VR Effect

**Figure 4:**
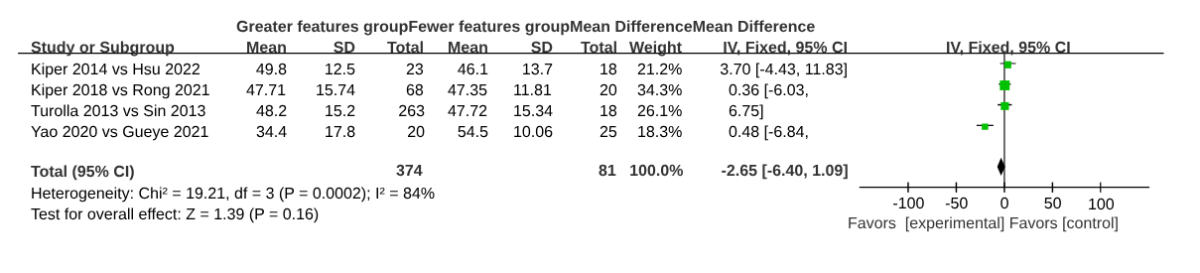
Forest plot of comparisons of VRT content design between the fewer and a greater number of feature subgroups. VRT: virtual reality therapy.

The content design factor was defined based on the National Clinical Guideline for Stroke for the United Kingdom and Ireland (2023 edition) [19]. The number of features in the VR interventions across all studies ranged from 2 to 10. Comparisons were made between studies with a fewer number of content features (n=7, features ≤5) and those with a greater number of features (n=8, features>5; Table 8). The common outcome variable among the included studies was upper limb function measured by the FMA-UE, and thus the comparisons were based on this measure. The MDs in FMA-UE scores did not significantly differ between the fewer and greater feature subgroups (MD 3.89, 95% CI –6.40 to 1.09; effect *Z*=1.36, *P*=.16; Figure 4; see also [11,42-46,48,50]).

**Table 8:** Content analysis of the design features of the VRT used in the studies.

Studies	Relative quantity of content features^a^	Content feature items									
		1^b^	2^c^	3^d^	4^e^	5^f^	6^g^	7^h^	8^i^	9^j^	10^k^
Brunner et al [40]	+	N/A^l^	✓^m^	✓	N/A	N/A	N/A	N/A	N/A	✓	N/A
Abd El-Kafy et al [41]	+++	✓	✓	✓	✓	✓	N/A	✓	N/A	N/A	N/A
Gueye et al [42]	+	N/A	✓	✓	N/A	N/A	N/A	N/A	✓	N/A	N/A
Hsu et al [11]	✓	N/A	✓	✓	N/A	N/A	N/A	N/A	N/A	N/A	N/A
Kiper et al [43]	+++	✓	✓	✓	✓	✓	✓	✓	✓	N/A	✓
Kiper et al [44]	+++	✓	✓	✓	✓	✓	✓	✓	✓	N/A	✓
Rong et al [45]	++	✓	✓	✓	N/A	N/A	N/A	✓	N/A	N/A	N/A
Schuster-Amft et al [16]	+++	✓	✓	✓	✓	✓	N/A	✓	✓	✓	N/A
Sin and Lee [46]	++	N/A	✓	✓	✓	✓	N/A	✓	N/A	N/A	N/A
Taveggia et al [47]	+	N/A	N/A	✓	✓	N/A	N/A	N/A	N/A	N/A	N/A
Yao et al [48]	+++	N/A	N/A	✓	✓	✓	✓	✓	N/A	N/A	✓
Zheng et al [49]	+++	✓	✓	✓	✓	✓	N/A	✓	✓	✓	N/A
Turolla et al [50]	+++	✓	✓	✓	N/A	✓	✓	✓	N/A	✓	N/A
Borstad et al [33]	+++	✓	✓	✓	✓	✓	✓	✓	✓	N/A	✓
Sebastián-Romagosa et al [34]	+	N/A	✓	✓	N/A	N/A	N/A	N/A	N/A	N/A	N/A

^a^The ‘+’, ‘++’, and ‘+++’ keys refer to a few (2-3), medium (4-5), and many (≥6) numbers of features, respectively.

^b^Item 1: Offering exercise and functional tasks.

^c^Item 2: Having individualized goals.

^d^Item 3: Quantifying activity.

^e^Item 4: Considering comorbidities and baseline activity level.

^f^Item 5: Incorporating patient’s needs.

^g^Item 6: Matching patient’s background such as education level.

^h^Item 7: Patient’s goal and interest directed.

^i^Item 8: Involving goal setting.

^j^Item 9: Progressive difficulty level.

^k^Item 10: Promoting self-efficacy.

^l^N/A: not applicable.

^m^The symbol ‘✓’ indicates that descriptions of the virtual reality intervention are found to contain the corresponding feature item.

## Discussion

### Principal Findings

This study aimed to examine the influence of patients’ age, delivery dosage and schedule, and content design on the outcomes of virtual reality interventions for patients with stroke. The meta-analytic results revealed that virtual reality training had significantly greater therapeutic effects than conventional training in improving upper limb function in patients with stroke. These effects included enhancements in upper limb function, as measured by the FMA-UE and ARAT, and improvements in activities of daily living, as measured by the FIM.

The demographic factor associated with more significant therapeutic effects was younger patient age compared with older age (mean 56.5 vs 65.9 years). Virtual reality training content factors contributing to greater effects included higher training dosage (>15 hours) delivered over 4-6 weeks and shorter sessions (mean 1.2 vs 2.0 hours) scheduled 4 or more times per week. Notably, shorter sessions (mean 1.2 hours) were linked to better outcomes in upper limb function, whereas longer sessions (mean 2.0 hours) were associated with improved outcomes in activities of daily living.

Our findings on the therapeutic effects of virtual reality training are largely consistent with those reported in 4 previous meta-analytic studies [9,36,54,55]. The consistent results confirm that virtual reality, as a novel and potentially useful technology, is effective in improving upper limb function and activities of daily living compared with conventional therapy after stroke. However, an inconsistent result was observed in the nonsignificant effects of virtual reality training on improving patients’ upper limb dexterity (measured by the BBT). This nonsignificant finding could be attributed to the smaller number of studies that used finger dexterity as an outcome variable (n=4) compared with those focusing on upper limb function or activities of daily living (n=8).

Younger patients with stroke demonstrated better outcomes than older patients in both upper limb function and activities of daily living. One plausible explanation is that younger patients may have been more motivated and actively engaged in the training, leading to better results. Previous studies have shown that increasing age is associated with lower participation in cognitively demanding activities [56]. Reduced participation inevitably results in lower gains from training. Spiteri et al [57] found that older adults (eg, 65-70 years) faced barriers such as mobility challenges, limited community accessibility, and insufficient guidance from health care professionals, which hindered their participation in physical activity (also see [58]). Our findings further emphasize the value of virtual reality training in poststroke rehabilitation. Addressing potential barriers, such as ensuring adequate supervision from health care professionals and improving accessibility, could enhance the effectiveness of VR-based training for older patients.

VR-based training offers immersive, imaginative, and interactive experiences [59,60] that facilitate functional recovery in patients with stroke [61,62]. These vivid experiences have been linked to participants engaging in more intensive cognitive and motor processes during training [63-66]. The cognitive processes involve bottom-up attention, which entails the encoding and processing of visual and auditory stimuli emitted in the virtual environment [67,68]. Top-down attention, by contrast, involves cognitive control, decision-making, and motor planning to produce motor responses [69,70]. The content design of virtual reality training did not significantly influence its therapeutic effects. In this study, we adhered to the principles outlined by the United Kingdom and Ireland for designing therapy aimed at promoting functional recovery in patients with stroke. The number of different features, whether fewer or more, based on these principles, did not emerge as a significant factor. However, 2 content features shared by most studies were setting individualized goals (item 2) and quantifying activities (item 3). Our findings suggest that counting repetitions of movements or responses performed by patients and setting treatment goals tailored to individual needs may be essential or sufficient features for designing effective virtual reality interventions.

The cognitive and motor processes involved in virtual reality training need to be repetitive to drive functional changes in patients with stroke. Our results suggest that more than 15 hours of training, scheduled over 4-6 weeks with 4 or more 1-hour sessions per week, can maximize improvements in upper limb function. By contrast, achieving significant gains in independence in activities of daily living appears to require a higher dosage, with 30 or more hours of training needed to produce meaningful outcomes. Repetitiveness is essential for initiating neural changes after a stroke. At the neurological level, posttraining changes in the motor cortices involve alterations in functional connectivity within neural networks, such as the prefrontal cortex and the basal ganglia [71,72]. Recovery of upper extremity function after a stroke has been linked to changes in functional connectivity between the bilateral primary motor cortex (M1) and the dorsolateral prefrontal cortex [73,74]. Additionally, repetitive training leads to the functional reorganization of the sensorimotor networks and the extrapyramidal system [75].

### Limitation

This study has several limitations. First, the subgroup analyses did not include outcomes measured by the ARAT or the WMFT, limiting the generalizability of the results. Caution is therefore needed when interpreting the findings. Second, the therapeutic effects of VR-based training were derived from outcomes measured shortly after the completion of training. As such, the findings do not extend to short- or long-term posttraining effects. Evidence regarding the long-term benefits of VR-enhanced exercise training for improving upper limb function in patients with stroke remains limited. However, a study examining the long-term treatment effects of VR in cardiovascular rehabilitation found that combining VR-based therapy with conventional treatment was associated with sustained long-term effects on hemodynamic and autonomic outcomes. This suggests that VR could serve as a valuable new treatment modality when integrated with cardiovascular rehabilitation sessions [51-53,76]. Standalone VR treatment lacks sensory and proprioceptive inputs to the central nervous system, limiting the functionality of the central-peripheral loop. Future longitudinal studies should explore the combination of VR-based therapy with actual exercises, focusing on how to integrate these approaches effectively for patients with stroke.

Third, significant heterogeneity (*I*^2^>50%) was observed in the meta-analysis. In the whole-group analysis, participants in the VRT group showed significantly greater improvement than those in the CON group, with *I*^2^<50%, indicating that the results from the studies were consistent in favoring VRT. However, in the within-group pre- and postintervention analysis, we found that some studies had *I*^2^>50%, such as the WMFT (*I*^2^=82%). This could be attributed to clinical variability in intervention delivery, including factors such as dosage, trial length, and frequency. The heterogeneity of participants across the included trials likely reduced the effect sizes of the observed therapeutic effects, particularly for older patients and improvements in finger dexterity. Similarly, the relatively small number of studies in the subgroup analyses may have reduced the power to identify training design content as a significant outcome factor. Future research should include more studies and patients with stroke in the analyses and investigate the long-term effects of virtual reality training.

### Conclusions

Virtual reality training was effective in promoting upper limb function and independence in activities of daily living for patients with stroke. Patient age and the dosage and schedule of training delivery are factors that influence its therapeutic effects. Younger patients who participate in training for more than 15 hours tend to show better outcomes compared with older patients. Training content delivered in 1-hour sessions, with more than 4 sessions per week over 4-6 weeks, could further maximize the therapeutic effects. Additionally, the design of the VR content should be tailored to the individual needs of the patients and involve them in setting treatment goals.

## Appendix

Multimedia Appendix 1 Search strategy.

Multimedia Appendix 2 PRISMA (Preferred Reporting Items for Systematic Reviews and Meta-Analyses) checklist.

## Abbreviations

ARAT: Action Research Arm Test
BBT: Box and Block Test
CON: conventional therapy
FIM: Functional Independence Measure
FMA-UE: Upper Extremity Fugl-Meyer Assessment
MD: mean difference
MINORS: Methodological Index for Non-Randomized Studies
PEDro: Physiotherapy Evidence Database
PRISMA: Preferred Reporting Items for Systematic Reviews and Meta-Analyses
RCT: randomized controlled trial
VR: virtual reality
VRT: virtual reality therapy
WMFT: Wolf Motor Function Test

## Data Availability

All data generated or analyzed during this study are included in this published article. Further inquiries can be directed to the corresponding author.
