# Supplementary material for: Virtual Reality Enhanced Exercise Training in Upper Limb Function of Patients With Stroke: Meta-Analytic Study
Source: J Med Internet Res. 2025 Feb 19;27:e66802. doi: 10.2196/66802 (PMC11888021; doi:10.2196/66802)
Supplement: Multimedia Appendix 1 [file jmir_v27i1e66802_app1.pdf]

Appendix. Search Strategy

PubMed (187 articles were retrieved)

#1 ((((((((((((((((((((((((((((((((("Paresis"[Mesh]) OR (Pareses)) OR (Muscular Paresis)) OR (Muscular Pareses)) OR (Pareses, Muscular)) OR (Paresis, Muscular)) OR (Muscle Paresis)) OR (Muscle Pareses)) OR (Pareses, Muscle)) OR (Paresis, Muscle)) OR (Monoparesis)) OR (Monopareses)) OR (Lower Extremity Paresis)) OR (Extremity Pareses, Lower)) OR (Extremity Paresis, Lower)) OR (Lower Extremity Pareses)) OR (Pareses, Lower Extremity)) OR (Paresis, Lower Extremity)) OR (Crural Paresis)) OR (Crural Pareses)) OR (Pareses, Crural)) OR (Paresis, Crural)) OR (Upper Extremity Paresis)) OR (Extremity Pareses, Upper)) OR (Extremity Paresis, Upper)) OR (Pareses, Upper Extremity)) OR (Paresis, Upper Extremity)) OR (Upper Extremity Pareses)) OR (Brachial Paresis)) OR (Brachial Pareses)) OR (Pareses, Brachial)) OR (Paresis, Brachial)) OR (Hemiparesis)) OR (Hemipareses)

#2 (((((((((((((((((((((((((((("Hemiplegia"[Mesh]) OR (Hemiplegias)) OR (Hemiplegia, Transient)) OR (Hemiplegias, Transient)) OR (Transient Hemiplegia)) OR (Transient Hemiplegias)) OR (Monoplegia)) OR (Monoplegias)) OR (Hemiplegia, Post-Ictal)) OR (Hemiplegia, Post Ictal)) OR (Hemiplegias, Post-Ictal)) OR (Post-Ictal Hemiplegia)) OR (Post-Ictal Hemiplegias)) OR (Hemiplegia, Crossed)) OR (Crossed Hemiplegia)) OR (Crossed Hemiplegias)) OR (Hemiplegias, Crossed)) OR (Hemiplegia, Flaccid)) OR (Flaccid Hemiplegia)) OR (Flaccid Hemiplegias)) OR (Hemiplegias, Flaccid)) OR (Hemiplegia, Infantile)) OR (Hemiplegias, Infantile)) OR (Infantile Hemiplegia)) OR (Infantile Hemiplegias)) OR (Hemiplegia, Spastic)) OR (Hemiplegias, Spastic)) OR (Spastic Hemiplegia)) OR (Spastic Hemiplegias)

#3 (((((("motor skills disorders "[Mesh]) OR (Motor Skills Disorder)) OR (Developmental Coordination Disorder)) OR (Coordination Disorder, Developmental)) OR (Developmental Coordination Disorders)) OR ("Motor Disorders"[Mesh])) OR (motor dysfunction*)

#4 #1 OR #2 OR #3

#5 (((((((("Upper Extremity"[Mesh]) OR (Extremities, Upper)) OR (Upper Extremities)) OR (Membrum superius)) OR (Upper Limb)) OR (Limb, Upper)) OR (Limbs, Upper)) OR (Upper Limbs)) OR (Extremity, Upper)

#6 (((((((((((("Virtual Reality"[Mesh]) OR (Reality, Virtual)) OR (Virtual Reality, Educational)) OR (Educational Virtual Realities)) OR (Educational Virtual Reality))

OR (Reality, Educational Virtual)) OR (Virtual Realities, Educational)) OR (Virtual Reality, Instructional)) OR (Instructional Virtual Realities)) OR (Instructional Virtual Reality)) OR (Realities, Instructional Virtual)) OR (Reality, Instructional Virtual)) OR (Virtual Realities, Instructional)

#7 ((((((("Augmented Reality"[Mesh]) OR (Augmented Realities)) OR (Realities, Augmented)) OR (Reality, Augmented)) OR (Mixed Reality)) OR (Mixed Realities)) OR (Realities, Mixed)) OR (Reality, Mixed)

#8 #6 OR #7

#9 #4 AND #5 AND #8

Embase (123 articles were retrieved)

#1 'paresis':ab,ti OR 'pareses':ab,ti OR 'muscular paresis':ab,ti OR 'muscular pareses':ab,ti OR 'pareses,muscular':ab,ti OR 'paresis,muscular':ab,ti OR 'muscle paresis':ab,ti OR 'muscle pareses':ab,ti OR 'pareses,muscle':ab,ti OR 'paresis,muscle':ab,ti OR 'monoparesis':ab,ti OR 'monopareses':ab,ti OR 'lower extremity paresis':ab,ti OR 'extremity pareses,lower':ab,ti OR 'extremity paresis, lower':ab,ti OR 'lower extremity pareses':ab,ti OR 'pareses, lower extremity':ab,ti OR 'paresis, lower extremity':ab,ti OR 'crural paresis':ab,ti OR 'crural pareses':ab,ti OR 'pareses, crural':ab,ti OR 'paresis, crural':ab,ti OR 'upper extremity paresis':ab,ti OR 'extremity pareses,upper':ab,ti OR 'extremity paresis,upper':ab,ti OR 'pareses,upper extremity':ab,ti OR 'paresis, upper extremity':ab,ti OR 'upper extremity pareses':ab,ti OR 'brachial paresis':ab,ti OR 'brachial pareses':ab,ti OR 'pareses, brachial':ab,ti OR 'paresis, brachial':ab,ti OR 'hemiparesis':ab,ti OR 'hemipareses':ab,ti

#2 'hemiplegia':ab,ti OR 'hemiplegias':ab,ti OR 'hemiplegia, transient':ab,ti OR 'hemiplegias, transient':ab,ti OR 'transient hemiplegia':ab,ti OR 'transient hemiplegias':ab,ti OR 'monoplegia':ab,ti OR 'monoplegias':ab,ti OR 'hemiplegia, post-ictal':ab,ti OR 'hemiplegia,post ictal':ab,ti OR 'hemiplegias, post-ictal':ab,ti OR 'post-ictal hemiplegia':ab,ti OR 'post-ictal hemiplegias':ab,ti OR 'hemiplegia, crossed':ab,ti OR 'crossed hemiplegia':ab,ti OR 'crossed hemiplegias':ab,ti OR 'hemiplegias, crossed':ab,ti OR 'hemiplegia, flaccid':ab,ti OR 'flaccid hemiplegia':ab,ti OR 'flaccid hemiplegias':ab,ti OR 'hemiplegias, flaccid':ab,ti OR 'hemiplegia, infantile':ab,ti OR 'hemiplegias, infantile':ab,ti OR 'infantile hemiplegia':ab,ti OR 'infantile hemiplegias':ab,ti OR 'hemiplegia, spastic':ab,ti OR 'hemiplegias, spastic':ab,ti OR 'spastic hemiplegia':ab,ti OR 'spastic hemiplegias':ab,ti

#3 'motor skills disorders':ab,ti OR 'motor skills disorder':ab,ti OR 'developmental coordination disorder':ab,ti OR 'coordination disorder,

developmental':ab,ti OR 'developmental coordination disorders':ab,ti OR 'motor disorders':ab,ti OR 'motor dysfunction*':ab,ti
#4 #1 OR #2 OR #3
#5 'upper extremity':ab,ti OR 'extremities, upper':ab,ti OR 'upper extremities':ab,ti OR 'membrum superius':ab,ti OR 'upper limb':ab,ti OR 'limb, upper':ab,ti OR 'limbs, upper':ab,ti OR 'upper limbs':ab,ti OR 'extremity, upper':ab,ti
#6 'virtual reality':ab,ti OR 'reality, virtual':ab,ti OR 'virtual reality, educational':ab,ti OR 'educational virtual realities':ab,ti OR 'educational virtual reality':ab,ti OR 'reality, educational virtual':ab,ti OR 'virtual realities, educational':ab,ti OR 'virtual reality, instructional':ab,ti OR 'instructional virtual realities':ab,ti OR 'instructional virtual reality':ab,ti OR 'realities, instructional virtual':ab,ti OR 'reality, instructional virtual':ab,ti OR 'virtual realities, instructional':ab,ti
#7 'augmented reality':ab,ti OR 'augmented realities':ab,ti OR 'realities, augmented ':ab,ti OR 'reality, augmented ':ab,ti OR 'mixed reality':ab,ti OR 'mixed realities ':ab,ti OR ' realities, mixed ':ab,ti OR 'reality, mixed ':ab,ti
#8 #6 OR #7
#9 #4 AND #5 AND #8

Web of Science (291 articles were retrieved)

TS=(Paresis OR Pareses OR Muscular Paresis OR Muscular Pareses OR Pareses, Muscular OR Paresis, Muscular OR Muscle Paresis OR Muscle Pareses OR Pareses, Muscle OR Paresis, Muscle OR Monoparesis OR Monopareses OR Lower Extremity Paresis OR Extremity Pareses, Lower OR Extremity Paresis, Lower OR Lower Extremity Pareses OR Pareses, Lower Extremity OR Paresis, Lower Extremity OR Crural Paresis OR Crural Pareses OR Pareses, Crural OR Paresis, Crural OR Upper Extremity Paresis OR Extremity Pareses, Upper OR Extremity Paresis, Upper OR Pareses, Upper Extremity OR Paresis, Upper Extremity OR Upper Extremity Pareses OR Brachial Paresis OR Brachial Pareses OR Pareses, Brachial OR Paresis, Brachial OR Hemiparesis OR Hemipareses OR Hemiplegia OR Hemiplegias OR Hemiplegia, Transient OR Hemiplegias, Transient OR Transient Hemiplegia OR Transient Hemiplegias OR Monoplegia OR Monoplegias OR Hemiplegia, Post-Ictal OR Hemiplegia, Post Ictal OR Hemiplegias, Post-Ictal OR Post-Ictal Hemiplegia OR Post-Ictal Hemiplegias OR Hemiplegia, Crossed OR Crossed Hemiplegia OR Crossed Hemiplegias OR Hemiplegias, Crossed OR Hemiplegia, Flaccid OR Flaccid Hemiplegia OR Flaccid Hemiplegias OR Hemiplegias, Flaccid OR Hemiplegia, Infantile OR Hemiplegias, Infantile OR Infantile Hemiplegia OR Infantile Hemiplegias OR Hemiplegia, Spastic OR Hemiplegias, Spastic OR Spastic Hemiplegia OR Spastic Hemiplegias OR motor skills disorders OR Motor Skills Disorder OR Developmental

Coordination Disorder OR Coordination Disorder, Developmental OR Developmental Coordination Disorders OR Motor Disorders OR motor dysfunction*) AND TS=(Upper Extremity OR Extremities, Upper OR Upper Extremities OR Membrum superius OR Upper Limb OR Limb, Upper OR Limbs, Upper OR Upper Limbs OR Extremity, Upper) AND TS=(Virtual Reality OR Reality, Virtual OR Virtual Reality, Educational OR Educational Virtual Realities OR Educational Virtual Reality OR Reality, Educational Virtual OR Virtual Realities, Educational OR Virtual Reality, Instructional OR Instructional Virtual Realities OR Instructional Virtual Reality OR Realities, Instructional Virtual OR Reality, Instructional Virtual OR Virtual Realities, Instructional OR Augmented Reality OR Augmented Realities OR Realities, Augmented OR Reality, Augmented OR Mixed Reality OR Mixed Realities OR Realities, Mixed OR Reality, Mixed)

the Cochrane Library (144 articles were retrieved)

#1 MeSH descriptor: [Virtual Reality] explode all trees

#2 Reality, Virtual or Virtual Reality, Educational or Educational Virtual Realities or Educational Virtual Reality or Reality, Educational Virtual or Virtual Realities, Educational or Virtual Reality, Instructional or Instructional Virtual Realities or Instructional Virtual Reality or Realities, Instructional Virtual or Reality, Instructional Virtual or Virtual Realities, Instructional： ti,ab,kw

#3 MeSH descriptor: [Augmented Reality] explode all trees

#4 Augmented Reality or Augmented Realities or Realities, Augmented or Reality, Augmented or Mixed Reality or Mixed Realities or Realities, Mixed or Reality, Mixed

#5 #1 or #2 or #3 or #4

#6 MeSH descriptor: [Motor Skills Disorders] explode all trees

#7 Motor Skills Disorder or Developmental Coordination Disorder or Coordination Disorder, Developmental or Developmental Coordination Disorders or Motor Disorders or motor dysfunction*:ti,ab,kw

#8 MeSH descriptor: [Paresis] explode all trees

#9 Pareses or Muscular Paresis or Muscular Pareses or Pareses, Muscular or Paresis, Muscular or Muscle Paresis or Muscle Pareses or Pareses, Muscle or Paresis, Muscle or Monoparesis or Monopareses or Lower Extremity Paresis or Extremity Pareses, Lower or Extremity Paresis, Lower or Lower Extremity Pareses or Pareses, Lower Extremity or Paresis, Lower Extremity or Crural Paresis or Crural Pareses or Pareses, Crural or Paresis, Crural or Upper Extremity Paresis or Extremity Pareses,

Upper or Extremity Paresis, Upper or Pareses, Upper Extremity or Paresis, Upper Extremity or Upper Extremity Pareses or Brachial Paresis or Brachial Pareses or Pareses, Brachial or Paresis, Brachial or Hemiparesis or Hemipareses:ti,ab,kw

#10 MeSH descriptor: [Hemiplegia] explode all trees

#11 Hemiplegias or Hemiplegia, Transient or Hemiplegias, Transient or Transient Hemiplegia or Transient Hemiplegias or Monoplegia or Monoplegias or Hemiplegia, Post-Ictal or Hemiplegia, Post Ictal or Hemiplegias, Post-Ictal or Post-Ictal Hemiplegia or Post-Ictal Hemiplegias or Hemiplegia, Crossed or Crossed Hemiplegia or Crossed Hemiplegias or Hemiplegias, Crossed or Hemiplegia, Flaccid or Flaccid Hemiplegia or Flaccid Hemiplegias or Hemiplegias, Flaccid or Hemiplegia, Infantile or Hemiplegias, Infantile or Infantile Hemiplegia or Infantile Hemiplegias or Hemiplegia, Spastic or Hemiplegias, Spastic or Spastic Hemiplegia or Spastic Hemiplegias:ti,ab,kw

#12 #6 or #7 or #8 or #9 or #10 or #11

#13 MeSH descriptor: [Upper Extremity] explode all trees

#14 Extremities, Upper or Upper Extremities or Membrum superius or Upper Limb or Limb, Upper or Limbs, Upper or Upper Limbs or Extremity, Upper:ti,ab,kw

#15 #13 or #14

#16 #5 and #12 and #15
